# A Few Large Roads or Many Small Ones? How to Accommodate Growth in Vehicle Numbers to Minimise Impacts on Wildlife

**DOI:** 10.1371/journal.pone.0091093

**Published:** 2014-03-19

**Authors:** Jonathan R. Rhodes, Daniel Lunney, John Callaghan, Clive A. McAlpine

**Affiliations:** 1 School of Geography, Planning and Environmental Management, The University of Queensland, Brisbane, Queensland, Australia; 2 ARC Centre of Excellence for Environmental Decisions, The University of Queensland, Brisbane, Queensland, Australia; 3 NERP Environmental Decisions Hub, The University of Queensland, Brisbane, Queensland, Australia; 4 Office of Environment and Heritage New South Wales, Hurstville, New South Wales, Australia; 5 School of Biological Sciences, University of Sydney, Sydney, New South Wales, Australia; 6 Australian Koala Foundation, Brisbane, Queensland, Australia; The Centre for Research and Technology, Hellas, Greece

## Abstract

Roads and vehicular traffic are among the most pervasive of threats to biodiversity because they fragmenting habitat, increasing mortality and opening up new areas for the exploitation of natural resources. However, the number of vehicles on roads is increasing rapidly and this is likely to continue into the future, putting increased pressure on wildlife populations. Consequently, a major challenge is the planning of road networks to accommodate increased numbers of vehicles, while minimising impacts on wildlife. Nonetheless, we currently have few principles for guiding decisions on road network planning to reduce impacts on wildlife in real landscapes. We addressed this issue by developing an approach for quantifying the impact on wildlife mortality of two alternative mechanisms for accommodating growth in vehicle numbers: (1) increasing the number of roads, and (2) increasing traffic volumes on existing roads. We applied this approach to a koala (*Phascolarctos cinereus*) population in eastern Australia and quantified the relative impact of each strategy on mortality. We show that, in most cases, accommodating growth in traffic through increases in volumes on existing roads has a lower impact than building new roads. An exception is where the existing road network has very low road density, but very high traffic volumes on each road. These findings have important implications for how we design road networks to reduce their impacts on biodiversity.

## Introduction

With landscapes becoming increasingly dominated by humans, the impact of roads on wildlife populations is growing rapidly [1], [2]. The direct effects of roads are wide ranging and include the destruction and modification of habitat [2], the modification of animal behaviour [3], the fragmentation of habitat by the formation of barriers [4], [5] and vehicle collisions [6]. Roads also indirectly affect wildlife populations by increasing human access to previously inaccessible areas and changing land use patterns [2], [7]. Consequently, understanding the impact and the conservation management implications of roads is a high priority for a wide range of species of conservation concern and for concerned scientists [8].

A particularly important impact associated with roads is elevated mortality rates from vehicle collisions. This has been shown to have substantial impacts on wildlife populations [6], [9], [10]. Fahrig et al. [11] show that the density of anurans (frogs) decreases and the proportion of dead anurans increases with traffic intensity on roads near Ottawa, Canada. They conclude that high traffic volumes increase mortality enough to significantly reduce population densities. Similarly, Jones [12] demonstrates the extinction of a population of eastern quolls (*Dasyurus viverrinus*) in Tasmania, Australia following a road upgrade. She links this to an increase in vehicle collision mortality due to higher vehicle speeds. Therefore, management of the mortality effects of roads is a critical consideration for the conservation of the ever-increasing proportion of biodiversity that occurs in close proximity to human settlements.

A major challenge for mitigating the impact of road mortality on wildlife is that, in almost all parts of the world, the number of vehicles on roads is increasing rapidly, with consequent increases in ecological impacts [13], [14]. Despite the importance of managing vehicle growth in a way that minimises impacts on wildlife populations, we currently have little understanding about the consequences of alternative road network design strategies for limiting the impact of vehicles on wildlife populations. This is because the focus to date has been predominantly on either only quantifying impacts [15] and/or evaluating mitigation measures such as road overpasses/underpasses and fencing on existing road networks only [16]–[18]. Evaluations of the implications of alternative road network designs are much rarer [19]–[21]. It is therefore important that we address this gap by placing more emphasis on evaluating alternative road network designs if we are to make informed decisions about future road network design strategies.

In terms of planning road networks, there are essentially two key ways in which the growth in vehicle numbers can be accommodated: (1) by upgrading existing roads to carry higher volumes of traffic and/or (2) by increasing the number (or density) of roads in the network. However, these two strategies result in different spatial distributions of traffic and therefore contribute to wildlife mortality rates via two different processes. If we increase the density of roads, but keep the traffic volume on each road constant, higher mortality rates arise because the probability that an animal moving around the landscape will cross a road increases [6]. On the other hand, if we increase traffic volume on each road, but keep the density of roads constant, higher mortality rates arise because the probability that an animal crossing a road is hit by a vehicle increases [6]. Although, under either strategy, mortality rates increase, it is far from intuitive which one results in the lowest increase in mortality, because that will depend upon the relative impact on mortality of two quite different processes. For a decision-maker faced with a choice between the two strategies, it is therefore critically important to understanding which strategy is best, and under which circumstances.

Friar et al. [19] present one of the few examples where alternative road network designs are evaluated with respect to road density and the placement of roads in relation to habitat. However, they do not consider how their results vary with traffic volume. On the other hand, van Langevelde and Jaarsma [20] do explore road network design strategies that modify the spatial distribution of traffic volumes, but only on existing roads. Jaeger et al. [21] consider the impact of alternative road network designs, including one where traffic volume is concentrated along a single road, but they only explore this in simple artificial landscapes. Therefore, an explicit evaluation of road network design strategies that modify road density versus strategies that modify traffic volumes in real landscapes is a research priority.

Here we address this issue using a spatially-explicit simulation model to quantify the relative impact of changes in road density and/or traffic volume on mortality rates for a koala (*Phascolarctos cinereus*) population in eastern Australia. For koalas, mortality on roads can form a large component of overall mortality rates in many areas and it is considered to be one of the key threatening processes for this species [22]–[26]. We characterise model outputs using a statistical approximation and ask whether general principles emerge about the relative benefits of accommodating more traffic by increasing the density of roads (i.e., more roads) versus increasing traffic volume on existing roads (i.e., larger roads). We show that, under most circumstances, it is preferable to accommodate a greater number of vehicles by increasing the capacity of existing roads, rather than building new roads.

## Materials and Methods

We used an existing model of koala movement for the study area [27] and no ethics permits or permissions were required to undertake the study.

### Study Species

The koala is a folivorous and arboreal marsupial restricted to the eucalypt forests and woodlands of eastern and south-eastern Australia. Across its range, the koala feeds on a wide variety of tree species, predominantly from the genera *Eucalyptus* and *Corymbia*, but in any particular area, they show preferences for just a few species [28]–[32]. Koala habitat generally consists of the preferred food tree species in any area, although other factors, such as tree size and water availability, can also contribute to habitat quality [32]–[35]. Koalas occupy reasonably well defined home ranges and, although largely solitary, both male and female home ranges can overlap [30], [36], [37]. The key threats to the species are habitat loss and fragmentation, urbanisation, dog attacks, vehicle collisions, disease, bushfire and climate change [22], [23], [25], [26], [38]–[41]. Movements between trees, particularly in fragmented peri-urban areas, are usually made along the ground and this includes movements across roads.

### Study Area

**Figure 1:**
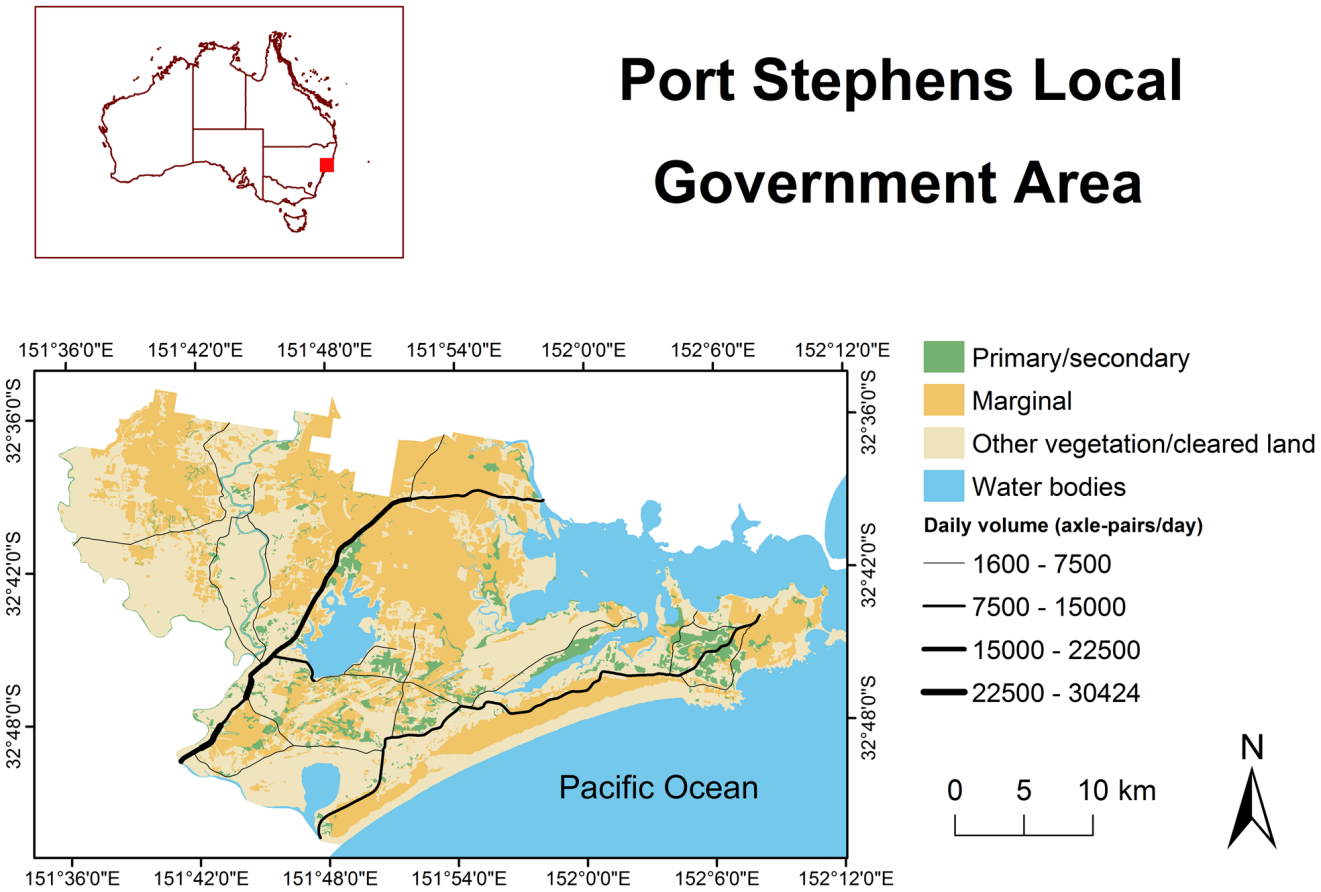
The Port Stephens Local Government Area. Map shows the study area's location in Australia, the estimated distribution of koala habitat, and the estimated average daily traffic volume (axle-pairs day^−1^) on major roads.

Our study area was located within the Port Stephens Local Government Area, New South Wales, Australia, approximately 150 km north of Sydney (Figure 1). This area contains one of the most significant koala populations in New South Wales [42]. However, since European settlement, habitat loss and fragmentation has occurred in the area due to agriculture, urbanisation and sand mining, bringing important threats to the koala population in the region [40], [41], [43]. Among these threats, vehicle collision mortality on roads is considered to be one of the most significant in Port Stephens [44].

### Simulation Model

To model the impact of roads on koala mortality, we combined a simulation model of koala movement with a model of the risk of mortality when crossing a road. Below we describe the model in detail and then describe how we applied it to our study area to quantify the relative impact of increases in road density versus increases in traffic volume.

#### Movement Model

We used a spatially-explicit habitat selection and movement model to simulate koala movements in the study area [27]. Movement was simulated on a raster grid, with a 50 m×50 m cell size, representing the distribution of koala habitat in Port Stephens. The spatial distribution of habitat was derived from an existing koala habitat model and vegetation maps for the area [27], [31], [33]. The habitat model was developed based on information on koala tree species preferences estimated from field surveys of koala faecal pellets and this was then combined with detailed vegetation maps of the study area to arrive at the final habitat map (see Appendix C in Rhodes et al. [27] for a full description of the habitat mapping procedures). Each raster cell was classified as either: (1) primary/secondary habitat; (2) marginal habitat; (3) other vegetation not classified as koala habitat; (4) cleared; or (5) water bodies (Figure 1). Rhodes et al. [27] separate primary and secondary habitat, but we combined these two habitat categories here to reduce the number of habitat classes [41]. For a detailed description of the habitat categories see Lunney et al. [33]. The spatial habitat data is available from the Dryad Digital Repository: http://dx.doi.org/10.5061/dryad.3n4h2.

Based on the movement model described in Rhodes et al. [27], we assumed that the probability of moving from location *a* to location *b*, Pr(*a* to *b*), in a given time period, in a landscape consisting of *k* = 1, …, *m* discrete grid cells, each of a defined habitat type *j* = 1, …, *n*, is
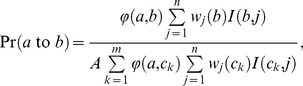
(1)where 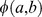 is a function defining the probability of moving from location *a* to location *b* independent of habitat (similarly for 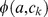); 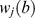 is the relative preference for habitat type *j* at location *b* (similarly for 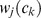); *A* is the area of each grid cell; *c_k_* is the location of the centre of grid cell *k*; and *I*(*b*,*j*) is an indicator function which equals 1 if the habitat at location *b* is of type *j* and equals 0 otherwise (similarly for *I*(*c_k_*,*j*)). The numerator defines the preference for moving from location *a* to *b* and the denominator is a normalisation constant that standardises this preference to a probability. The movement model represented by equation (1) is essentially a biased random-walk model with movement biased toward preferred habitat. It achieves this by modelling an underlying random-walk described by the function 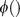, which defines the probability of movement to any location in the landscape in the absence of habitat selection, that is then modified by habitat preference through the function 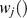. Note here that habitat preference is assumed to be dependent on the location of the habitat (see below).

The habitat independent movement probability function, 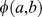, was defined as
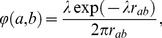
(2)where *λ* is the scale parameter for the negative-exponential distribution and *r_ab_* is the distance between location *a* and location *b*. This assumes that, in the absence of habitat selection, the probability distribution of movement steps is negative-exponential distributed. The habitat preference parameters, 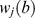, were defined as
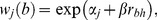
(3)where *α_j_* is the preference for habitat *j*; *r_bh_* is the distance from location *b* to the centre of the animal's home range; and the parameter β defines how habitat preference varies with distance from the home range centre [27]. Negative values for β imply a tendency to move back towards the centre of the home range and therefore introduce a form of home range behaviour. This model therefore enables the simulation of movement that accounts for both habitat selection and movement behaviour within a home range. For a full description of the movement model, habitat models and parameterisation see Rhodes et al. [27].

#### Road and Traffic Volume Data

Data on average daily traffic volumes (axle-pairs day^−1^) from traffic recording stations in Port Stephens between 1995 and 2001 (New South Wales Roads and Traffic Authority unpublished data, Port Stephens Council unpublished data) were used to estimate average daily traffic volumes on all the major roads in the study area. These traffic volumes were then mapped spatially (Figure 1). By linking traffic volumes to the spatial location of roads, spatial variation in traffic volumes were explicitly defined for incorporation into the model. The traffic volume spatial data is available from the Dryad Digital Repository: http://dx.doi.org/10.5061/dryad. 3n4h2.

For some traffic recording stations we also had data on hourly road traffic volumes from 2001 (New South Wales Roads and Maritime Services unpublished data) and this revealed a dramatic difference in traffic volumes between day and night. Koalas are more active during the night-time than during the day [36], [45], so the vast majority of road crossings will occur during the night. Therefore, we were interested in quantifying the proportion of traffic volumes that occur between 1800 h and 0600 h. We characterised this by fitting a beta distribution, by maximum-likelihood, to the proportion of daily traffic volumes occurring in each hour between 1800 h and 0600 h for the roads where we had hourly volume data. This provided estimates for the rate parameter, *r*, and scale parameter, *s*, for the beta distribution, beta(*r*, *s*) [46]. We estimated *r* = 1.32 and *s* = 87.16 and subsequently used this distribution, combined with the average daily traffic volumes, to draw random values for hourly traffic volumes on each road during the times when koalas are most likely to cross roads.

#### Mortality Risk Model

To estimate the risk of mortality when a koala crosses a road, we used a simple model of the probability of being hit by a car. We assumed that the number of cars passing along a road per unit of time is Poisson distributed; a reasonable assumption for night-time vehicle volumes [47]. When a koala crosses the lane of a road, a gap between vehicles greater than the amount of time it takes the koala to cross the part of the road traversed by vehicles is required for a successful crossing. Therefore, assuming that koalas arrive randomly at a road and cross immediately, the probability of surviving a crossing is
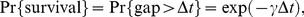
(4)where Δ*t* is the time taken to cross the part of the road traversed by vehicles and γ is the rate parameter for the Poisson distribution, representing the traffic volume. For a road with *n* lanes, with total two-way traffic volume, *γ*, and assuming traffic volume is divided equally between the lanes, i.e. 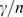 on each lane, then the probability of surviving a crossing of the entire road [48] is
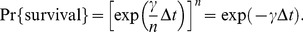
(5)


This general derivation holds for any *n*-laned road, provided vehicle volumes are split evenly among lanes [47].

We further assumed that all vehicles on each lane travel along the same part of the road and that koalas cross perpendicular to the flow of traffic. In this case, the time taken to cross the path traversed by vehicles is
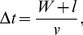
(6)where *W* is the vehicle width; *l* is the koala head to tail length; and *v* is the velocity at which koalas cross [47], [48]. Hels and Buchwald [9] take a similar approach, but include the possibility that road crossings occur at different angles and that the killing width of the car is only a proportion of the vehicle width. The subjects of their study were amphibians, for which individuals are often only hit if they are under the wheels of a vehicle. We made the reasonable assumption, given the size of a koala, that passing anywhere under a vehicle would result in a fatal collision. For simplicity, and in the absence of data on the distribution of crossing angles, we also assumed that all crossings are made perpendicular to the flow of traffic.

#### Movement Model Parameter Estimates and Uncertainty

Model parameters for the movement model for males and females were estimated from a koala radio-tracking data set for the Tomago Sandbeds region of Port Stephens [27]. This provided estimates of: habitat preference for marginal habitat, *α_marg_*, other vegetation/mining revegetation, *α_other_*, cleared land, *α_clear_*, the negative-exponential scale parameter, *λ*, and the parameter determining the influence of the distance to the home range centre, *β_hr_* for each sex (Table 1). Habitat preference parameters were all estimated relative to primary/secondary habitat and water bodies were assumed unavailable. Head to tail lengths, *l*, were estimated as 0.66 m for females and 0.70 m for males using data for 69 adult female and 48 adult male koalas from South East Queensland (Table 1, Queensland Department of Environment and Heritage Protection unpublished data). The velocity, *v*, at which koalas cross roads is uncertain and, to our knowledge, no empirical data currently exists for this. In the absence of such information, we assumed that this parameter would be somewhere in the range 5000–15000 m h^−1^ (Table 1). These values are based on personal observations (D. Lunney) that koala movement velocities across roads would be at least as fast as a typical human walking speed of around 5000 m h^−1^, but could be higher. We assumed that the average width of vehicles, *W*, was 2 m (Table 1).

**Table 1:** Parameter values used in the simulations.

Parameter	Symbol	Baseline Value	Standard Error/Range
Females			
Habitat preference for marginal habitat	*α_marg_*	−0.210	0.057
Habitat preference for other vegetation	*α_other_*	−0.474	0.086
Habitat preference for cleared land	*α_clear_*	−0.717	0.118
Negative-exponential scale parameter	*λ*	4.77×10^−3^ m^−1^	0.1360×10^−3^
Distance to the home range centre parameter	*β_hr_*	−3.77×10^−3^ m^−1^	0.1466×10^−3^
Head to tail length	*l*	0.659 m	0.008
Movement velocity	*v*	10000 m h^−1^	5000–15000
Males			
Habitat preference for marginal habitat	*α_marg_*	−0.262	0.073
Habitat preference for other vegetation	*α_other_*	−0.396	0.120
Habitat preference for cleared land	*α_clear_*	−0.373	0.175
Negative-exponential scale parameter	*λ*	2.52×10^−3^	0.0928×10^−3^
Distance to the home range centre parameter	*β_hr_*	−2.52×10^−3^	0.1014×10^−3^
Head to tail length	*l*	0.700 m	0.012
Movement velocity	*v*	10000 m h^−1^	5000–15000

The habitat preference parameter for primary/secondary habitat was fixed at zero, so all habitat preference parameters are relative to primary/secondary habitat. *α_marg_*  =  habitat preference parameter for marginal habitat, *α_other_*  =  habitat preference parameter for other vegetation/mining revegetation and *α_clear_*  =  habitat preference parameter for cleared land.

We also characterised uncertainty in the estimates of the parameters *α_marg_*, *α_other_*, *α_clear_*, *λ*, *β_hr_*, *l* and *v*. We did this for the parameters *α_marg_*, *α_other_*, *α_clear_*, *λ* and *β_hr_* by assuming that they were distributed multivariate normal with expected values and variance-covariance matrix estimated from the log-likelihood of the movement model [27]. Koala head to tail lengths were also assumed to be normally distributed with means and variances derived from the sample means and standard errors of the head to tail length data [49]. Although we used sampling distributions, rather than Bayesian posterior distributions, to describe parameter uncertainty, when using uninformative priors, sampling distributions will tend to approximate the Bayesian posterior distributions [50], [51]. Therefore, these distributions were deemed to be adequate approximations for parameter uncertainties given the data underlying their estimation. The high degree of uncertainty in the velocity at which koalas cross roads, *v*, was characterised by considering three separate values for this parameter: 5000, 10000 and 15000 m h^−1^.

#### Simulations

For each simulation run, 500 male and 500 female koalas were located randomly on the landscape and their movements were simulated for 365 days in 24-hour time steps. We assumed that their locations at the start of the simulations were the centres of their home ranges (i.e., they started from their home range centres). For each road crossed during a koala movement, we determined whether a mortality event occurred or not. This was achieved by first drawing a random variable from the beta distribution describing the probability distribution of hourly proportions of traffic volumes between 1800 h and 0600 h. This random proportion was then multiplied by the average daily volume for the road crossed to obtain an hourly traffic volume at the time of the crossing, thus assuming that the time of crossing was chosen randomly between 1800 h and 0600 h. Assuming that one axle-pair is one vehicle, the probability of surviving the road crossing was calculated from equation (5) using the randomly drawn traffic volume. Whether the individual survived the crossing was based on a draw from a Bernoulli distribution with probability equal to the probability of surviving the road crossing. An individual that did not survive a road crossing was recorded as such, but movement simulations were continued for the entire 365 days so that an estimate of the individual's home range could subsequently be calculated.

The simulated daily locations for each individual were then used to construct a 95% fixed kernel home range using a smoothing parameter, *h*, equal to the resolution of the landscape of 50×50 m [52]. Within the estimated home range, the road density (i.e., the proportion of grid cells in the home range that contained a road), *X_dens_*, and the mean traffic volume (i.e., then mean road traffic volume in grid cells containing roads), *X_vol_*, were recorded.

### Statistical Analysis

We used the simulation outputs to develop logistic regression models of the risk of mortality due to vehicle collision as a function of road density and traffic volume [53]. The response variable for these models was the binary mortality/survival data for each individual, with *X_dens_*, *X_vol_* and a *X_dens_* by *X_vol_* interaction as explanatory variables. We excluded those individuals whose home ranges did not contain any roads. If there were no roads in an individual's home range, the mortality risk was always zero, so we were interested only in developing models for estimating the mortality risk, conditional on at least one road being present in an individual's home range. The regression models took the form
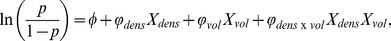
(7)where *p* is the probability of mortality and 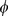, 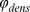, 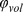 and 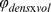 are the regression coefficients. Collinearity between mean traffic volumes and road densities was low based on Pearson's correlation coefficients (*ρ*<0.1) and therefore collinearity was not considered an issue for the regression models.

To validate the models, we first ran simulations for each of the three alternative values of movement velocity, *v*, with baseline parameter values otherwise, for each sex (Table 1). Logistic regression models were fitted to each of the six resulting simulated datasets and we tested the model fits using Hosmer-Lemeshow deciles of risk and Pearson *χ* ^2^ global goodness-of-fit tests [53]. The *p*-values for the Pearson *χ* ^2^ tests were calculated from a normal approximation of the statistic's distribution [53]–[55].

We then used a bootstrap approach to estimate the expected values and standard deviations of the logistic regression parameters so as to capture the uncertainty that arises from uncertainty in the simulation model parameters [56], [57]. For each sex, and for each of the three values of *v* (5000, 10000 and 15000 m h^−1^), we chose 100 random values of *α_marg_*, *α_other_*, *α_clear_*, *λ*, *β_hr_* and *l* from the distributions describing the uncertainty in these parameter values. For each combination of parameter values, we ran a set of simulations and fitted the logistic regression model to the data, as for the baseline parameter case. This was repeated for each value of *v* and, for each of these, the mean and standard deviation of the estimated regression model parameters from the 100 replicates were calculated. This bootstrap approach provided, for each value of *v*, an estimate of the expected value and standard deviation of the regression coefficients [58]. The standard deviations primarily reflect parameter uncertainty, which is propagated through the random draws from the parameter distributions, rather than simulation error. Although some simulation error will be present in these estimates, the large number of individuals simulated for each parameter combination (500 of each sex) means that simulation error is likely to be relatively small. Efron and Tibshirani [58] recommend that between 50 and 200 bootstrap replicates are usually required to reliably estimate standard deviations. Therefore, the 100 replicates we used in this study were sufficient to obtain reasonable estimates of the parameter means and standard deviations. The bootstrap expected values of the regression coefficients were then used to make predictions about the probability of mortality due to vehicle collision mortality for values of *X_dens_* between 0.002 and 0.2 and *X_vol_* between 30 and 30000 axle-pairs day^−1^.

Finally, we aimed to quantify the impact of changes in *X_dens_* and *X_vol_* on vehicle collision mortality risk. To do this, we calculated the sensitivity and the elasticity of 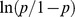 to changes in road density and traffic volume. Sensitivity with respect to road density, *s_dens_*, and traffic volume, *s_vol_*, were calculated, from equation (7), as
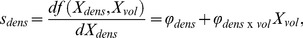
(8)and
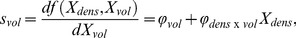
(9)where 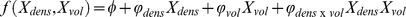 is the linear predictor in equation (7).

However, the values of road density and traffic volume were on quite different scales, making the relative interpretation of sensitivities difficult. As an alternative approach, elasticities provide a means of comparing the effect of proportional changes in variables, thus making them comparable. More specifically, elasticities provide a measure of the proportional change in one variable in response to a proportional change in another [59]. However, because the proportion of a logit has no intrinsic meaning, we modified the formulae for calculating elasticities so that we obtained the absolute change in 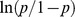 resulting from a proportional change in road density or traffic volume. In so doing, the elasticities with respect to road density, *e_dens_*, and traffic volume, *e_vol_*, were calculated as
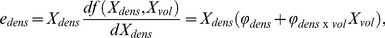
(10)and
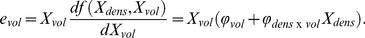
(11)


To compare relative elasticities, we used the ratio *e_dens_*/*e_vol_* and calculated this for *X_dens_* between 0.002 and 0.2 and *X_vol_* between 30 and 30000 axle-pairs day^−1^, using the bootstrap expected values of the regression coefficients. To investigate robustness to parameter uncertainty, we also calculated the sensitivity, elasticity and the ratio of elasticities for each bootstrap replicate at typical mean values of *X_dens_*  = 0.04 and *X_vol_*  = 9500 axle-pairs day^−1^. These values were then summarised by their expected values and standard deviations.

## Results

### Statistical Model Adequacy

There was no evidence of a significant lack of fit for any of the logistic regression models fitted to the simulated data for the baseline parameters values based on either the Hosmer-Lemeshow deciles of risk or the Pearson *χ* ^2^ global goodness-of-fit tests (*p*>0.05). Therefore, the logistic regression models were considered adequate descriptions of the relationship between mortality risk, road density and traffic volume.

### Regression Coefficients

The expected values of the logistic regression coefficients showed that the probability of a mortality event on a road was positively related to road density and traffic volume, as expected (Table 2). There was considerable variation in estimates due to the propagation of uncertainty in simulation model parameters, but a high proportion of parameter draws resulted in positive slopes. However, the effect of road density was positive more often than the effect of traffic volume; coefficients for roads density were positive between 93% and 100% of the time, while coefficients for traffic volume were positive between 72% and 83% of the time. The coefficients for the interaction term was also positive, indicating that the impact of changes in road density was greater when traffic volumes were high than when they were low and/or the impact of changes on traffic volume was greater when road density was high than when it was low (Table 2). Uncertainty in the simulation model parameters fed through to relatively high standard deviations (Table 2), but again, the proportion of parameter combinations that resulted in a positive interaction term was high (between 91% and 100%). Overall, positive coefficients occurred more often for males than for females.

**Table 2:** Expected values and standard deviations (in parentheses) of the logistic regression coefficients for each sex and each movement velocity, *v*.

v	Intercept	Density	Volume	Interaction
		(X_dens_)	(X_vol_)	(X_dens_ × X_vol_)
Females				
5000	−2.56 (0.71)	33.69 (17.85)	4.25×10^−5^ (6.89×10^−5^)	3.71×10^−3^ (2.35×10^−3^)
10000	−2.97 (0.75)	29.76 (17.98)	5.12×10^−5^ (6.13×10^−5^)	2.27×10^−3^ (1.91×10^−3^)
15000	−3.09 (0.78)	23.70 (15.53)	3.28×10^−5^ (6.65×10^−5^)	2.28×10^−3^ (1.54×10^−3^)
Males				
5000	−2.20 (0.55)	43.18 (20.55)	3.99×10^−5^ (5.14×10^−5^)	6.67×10^−3^ (3.17×10^−3^)
10000	−2.62 (0.52)	35.98 (15.77)	3.69×10^−5^ (4.42×10^−5^)	4.59×10^−3^ (1.90×10^−3^)
15000	−2.90 (0.58)	33.05 (18.42)	4.23×10^−5^ (4.41×10^−5^)	3.53×10^−3^ (1.75×10^−3^)

Values are the sample means and standard deviations of the parameter estimates for the 100 bootstrap replicates.

### Predictions

**Figure 2:**
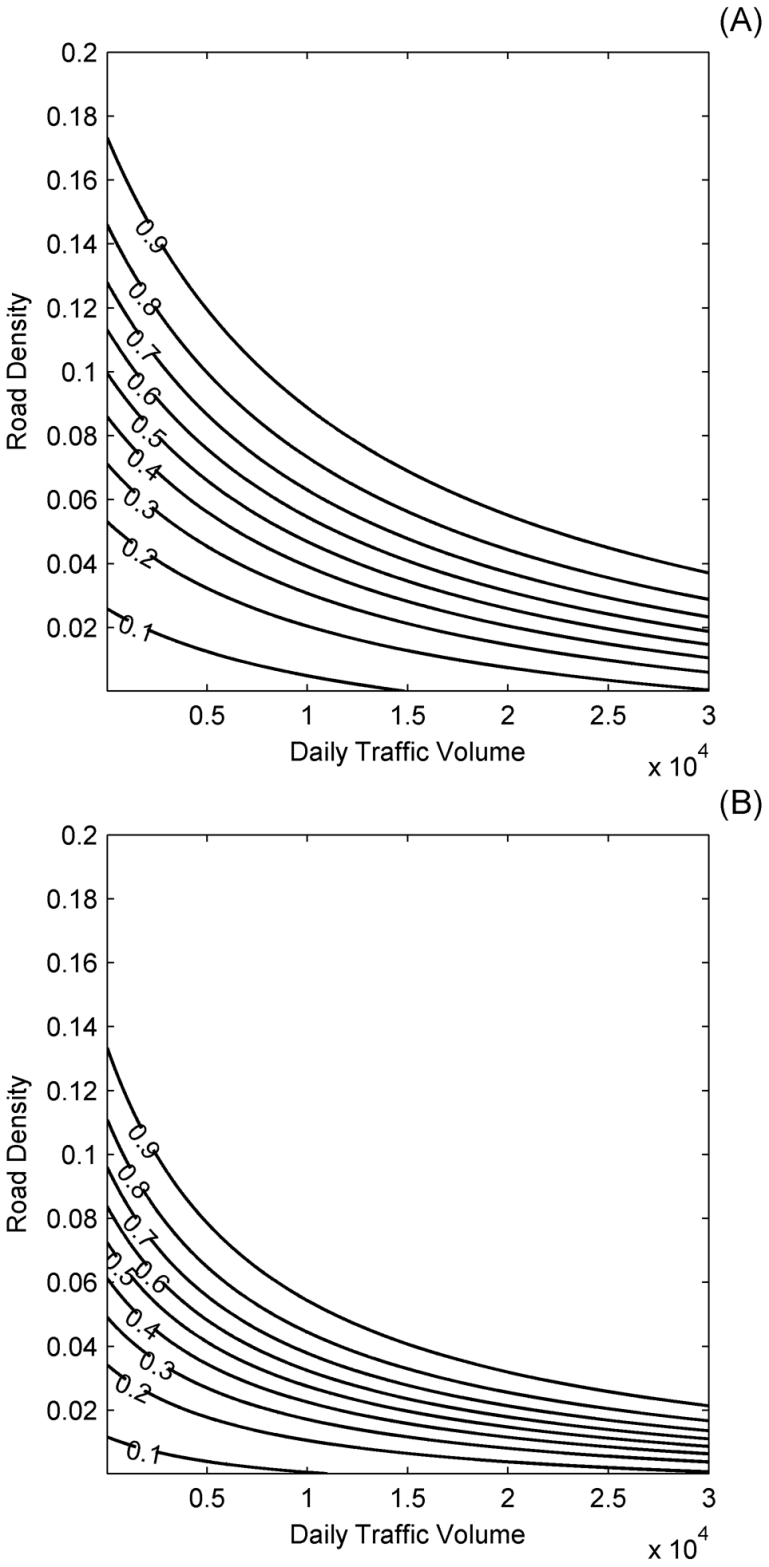
Contour plot of the predicted annual probability of mortality. Values are shown as a function of mean traffic volume, *X_vol_* (axle-pairs day^−1^) and road density, *X_dens_*>0 (proportion of grid cells containing a road) for: (A) females and (B) males. Annual probabilities of mortality were calculated from the bootstrap expected values of the regression coefficients with *v* = 10000 m h^−1^ (Table 2).

Predictions based on the regression models showed that males had higher annual mortality rates than females but, for both sexes, even low road densities were capable of causing high probabilities of mortality unless traffic volumes were very low (Figure 2). The major roads in Port Stephens have relatively high traffic volumes (the lowest recorded traffic volume on the roads used in the study was 1600 axle-pairs day^−1^ and the highest was over 30000 axle pairs day^−1^) indicating that, even areas with low road densities may impose high rates of mortality on koalas. Mortality rates increased with road density and traffic volume, but generally more rapidly with road density than traffic volume (Figure 2).

At typical mean values for road density (0.04) and traffic volume (9500 axle-pairs day^−1^), the sensitivities and elasticities were higher for males than females and declined as movement velocity, *v*, increased (Table 3). However the ratio of elasticities was similar across sexes and *v* and consistently showed a greater elasticity to proportional changes in road density than traffic volume. The elasticity with respect to road density was approximately 50% higher than for traffic volume. Bootstrap estimates of the sensitivities and elasticities were greater than zero 100% of the time, indicating strong support for an increase in mortality due to both increasing road density and traffic volume. There was slightly less strong support for the elasticity to road density being greater than the elasticity to traffic volume, but the ratio of elasticities was still greater than one for between 94% and 100% of parameter combinations.

**Table 3:** Expected values and standard deviations (in parentheses) of the sensitivities and elasticities of the logit probability of mortality with respect to road density, *s_dens_* and *e_dens_* and traffic volume, *s_vol_* and *e_vol_* and the ratio, *e_dens_*/*e_vol_*, for each sex and each movement velocity, *v*.

v	s_dens_	s_vol_	e_dens_	e_vol_	e_dens_/e_vol_
Females					
5000	68.93 (12.22)	1.91×10^−4^ (0.56×10^−4^)	2.76 (0.49)	1.81 (0.52)	1.60 (0.37)
10000	51.37 (10.44)	1.42×10^−4^ (0.39×10^−4^)	2.05 (0.42)	1.35 (0.37)	1.61 (0.46)
15000	45.36 (10.35)	1.24×10^−4^ (0.34×10^−4^)	1.81 (0.41)	1.18 (0.32)	1.69 (0.78)
Males					
5000	106.57 (18.99)	3.07×10^−4^ (0.95×10^−4^)	4.26 (0.76)	2.91 (0.91)	1.55 (0.38)
10000	79.57 (13.04)	2.20×10^−4^ (0.50×10^−4^)	3.18 (0.52)	2.09 (0.48)	1.57 (0.34)
15000	66.62 (11.35)	1.84×10^−4^ (0.43×10^−4^)	2.66 (0.45)	1.74 (0.41)	1.60 (0.43)

Sensitivities and elasticities were calculated at a typical mean road density of 0.04 and a typical mean traffic volume of 9500 axle-pairs day^−1^. Values are the sample means and standard deviations of the sensitivities and elasticities for the 100 bootstrap replicates.

**Figure 3:**
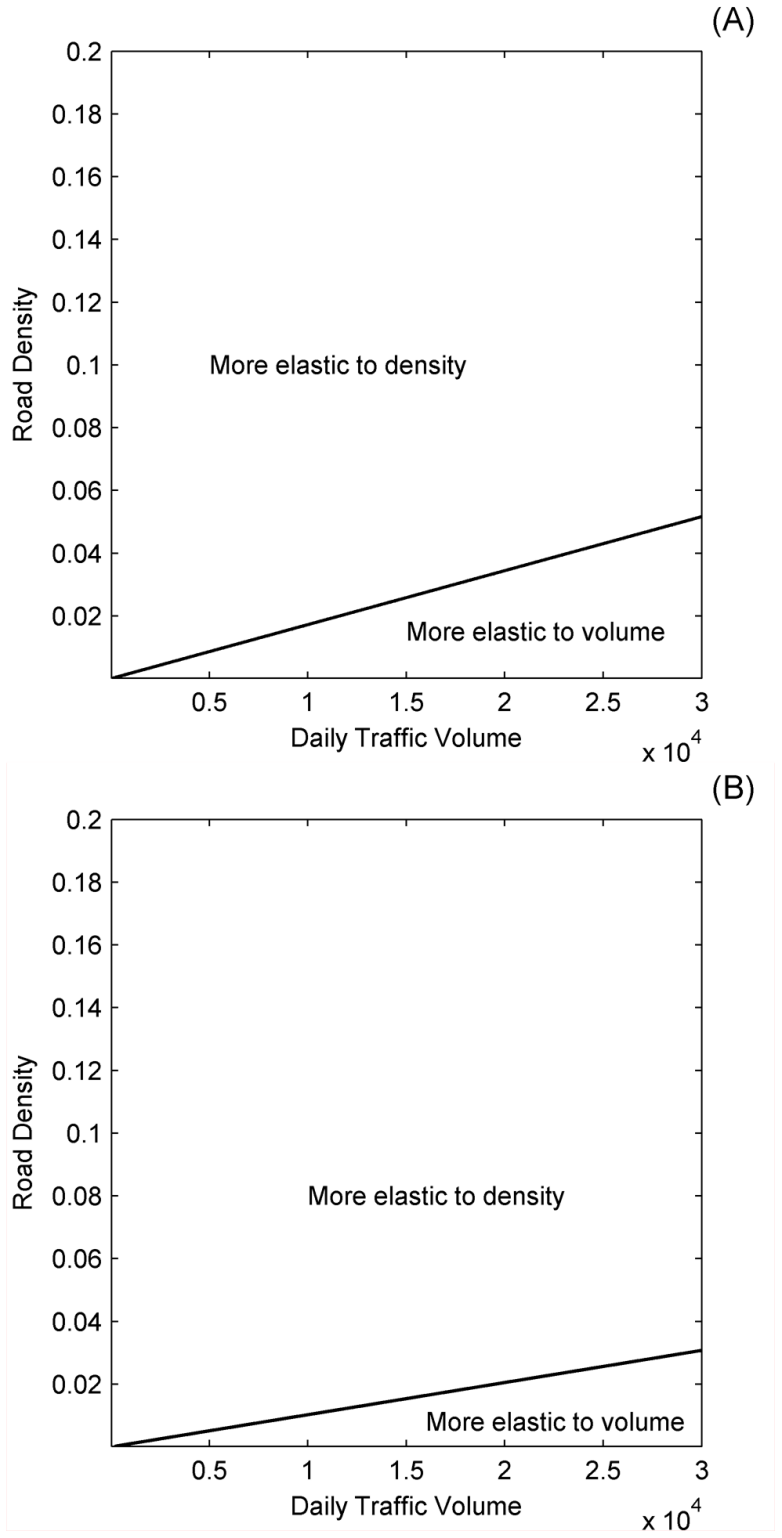
Plot of the regions where the annual probability of mortality is more elastic to road density and where the annual probability of mortality is more elastic to traffic volume. These regions are shown as a function of mean traffic volume, *X_vol_* (axle-pairs day^−1^) and road density, *X_dens_*>0 (proportion of grid cells containing a road) for: (A) females and (B) males. Elasticities were calculated from the bootstrap expected values of the regression coefficients with *v* = 10000 m h^−1^ (Table 2).

The elasticity ratio across the range of different values of road density and traffic volumes showed that, for most of the road density and traffic volume state-space, mortality was more elastic to a proportional change in road density, *X_dens_*, than the same proportional change in traffic volume, *X_vol_* (Figure 3). The exception was when road density was very low and traffic volume was very high, in which case there was a greater sensitivity to traffic volume. This general pattern held across all three values of movement velocity, *v*, and for both sexes, but especially for males.

## Discussion

As the world becomes increasingly urbanised and human population sizes increase, identifying strategies to accommodate these changes while limiting impacts on biodiversity is critical [60], [61]. Increases in road density and traffic volumes are typical features associated with greater urbanisation and human population growth, and are therefore fundamental considerations in conserving biodiversity [62]. A key planning decision that needs to be made for reducing the impact of roads on wildlife is whether we should accommodate increased traffic loads by increasing traffic on existing roads, by increasing the density of roads, or through a combination of both [63]. We have shown that, by using a simple model of animal movement and road mortality, key insights can be gained about the relative impacts of increases in road density versus increases in traffic volume on existing roads. In the vast majority of cases, we found that increasing road density elevated mortality rates more rapidly than did increasing traffic volume on existing roads. Decisions about where and how to build additional road capacity are governed by a range of factors, such as the spatial distribution of capacity requirements and implications for congestion [63]. However, our study indicates that strategies that focus on the creation of new roads are likely to be more harmful to wildlife than those that build capacity within an existing network.

Previous studies have demonstrated that both road density and traffic volume can have substantial impacts on mortality rates and population dynamics [6], but we still know comparatively little about the relative impact of changes in road density versus traffic volume. Our most important new insight here is that the effect of road density on mortality is commonly higher than the effect of traffic volume. The opposite was only true when road density was very low and traffic volume very high. Therefore, it is only in cases where the existing road network is characterised by very few high capacity roads that increases in network capacity by building new roads is likely to have the least impact on koala mortality. The model also indicated that male koalas were more susceptible to road traffic mortality than females and the range of conditions under which building new roads was the better strategy was even more limited for males than females. The reason for this is that males generally have larger home ranges and move greater distances than females, particularly during the breeding season, with the result that males tend to cross roads more frequently than females. This is consistent with empirical evidence on differences between sexes in vehicle collision mortality rates in koalas [22], [23]. The higher movement rates of male koalas also makes them more susceptible to the effect of increased road densities versus increased traffic volumes on existing roads. Therefore, in the case of males, this further reduces the range of conditions under which it is preferable to increase road network capacity by building new roads. The more general implications of this are that, for mobile species, upgrading existing roads is even more likely to be the better strategy than it is for less mobile species.

Despite uncertainty in the parameter estimates for the regression models, the sensitivities and elasticities of mortality with respect to road density and traffic volume were greater than zero for all simulation model parameter combinations. Although we are slightly more uncertain about the relative effects of increases in road density versus increases in traffic volume on existing roads, mortality is still more elastic to road density than traffic volume for the vast majority of the simulation model parameter combinations. Therefore, even after accounting for parameter uncertainty, the conclusion that accommodating increased traffic through higher volumes on existing roads has a lower impact on mortality than accommodating increased traffic through the building of new roads is relatively robust.

Although we focus on a single species here, the design of road networks will most commonly need to consider impacts on multiple species. Therefore, the extent to which our results can be generalised to other species is an important consideration. For example, species differ in their avoidance responses to roads, their movement speeds and their visibility to motorists and these factors may influence the relative impact of road density versus traffic volume [47], . We have shown that our conclusions are robust to movement speed, but we did not consider how avoidance behaviour or visibility to drivers could influence our results.

Behavioural responses to roads have been observed for a range of species, particularly avoidance behaviour [2]. Avoidance of roads varies among species and can depend on a range of factors such as, the size of roads, traffic volumes and road noise [66]–[69]. Further, avoidance can be an important factor determining road mortality and may therefore modify the relative effects of road density and traffic volume on mortality [64]. Jaeger et al. [21] relate road avoidance behaviour to traffic density in an artificial landscape and show that the impact of roads on persistence is lower when traffic volumes are concentrated along a single road than when they are not. Although they only consider an artificial landscape, their results are consistent with ours across a range of traffic volumes and avoidance probabilities. This indicates that our results may be robust to assumptions about avoidance behaviour. In our model we also assume that drivers do not respond to animals on roads by taking evasive action, with this essentially being equivalent to assuming that the visibility of animals is zero. Incorporating visibility and the possibility of drivers taking evasive action would increase the probability of survival and would have an analogous effect to reducing traffic volumes. This effect would make it more likely for road density to have a larger impact that traffic volumes (Figure 3). Therefore, our results are also likely to be robust to variation in visibility across species.

We quantified the relative impact on mortality from increases in traffic volume on existing roads and from increasing road density, but did not explicitly consider the spatial drivers of these patterns. The effect of each of these will depend upon the spatial locations of existing roads and the potential locations of any future roads relative to habitat. Where roads occur in close proximity to habitat or resources required by species, then this can result in the attraction of wildlife to roads, resulting in higher mortality rates [70], [71]. Friar et al. [19] show that the road mortality hazard for elk (*Cervus elephus*) is higher for roads associated with clearcuts than for roads independent of clearcuts. Therefore, the location of new roads is likely to be a critical factor in determining the actual impact on mortality. If new roads are necessary to accommodate increased traffic, then the impact on wildlife may be reduced by avoiding locations close to existing habitat. An alternative and potentially complementary strategy that has been proposed for reducing road impacts involves traffic calming in key habitat areas and redirecting traffic to surrounding roads [20], [48]. For example, van Langevelde and Jaarsma [20] show that traffic calming results in improvements in roe deer (*Capreolus capreolus*) persistence by reducing fragmentation effects. The mechanism underlying the success of this strategy is similar to the mechanism that drives the relative success of accommodating increased vehicle numbers by raising capacity on existing roads versus building new roads; it reduces the number of high-volume roads that individual animals need to cross. However, the effect of traffic calming is also likely to depend critically on the spatial distribution of both habitat and the selection of areas for traffic calming. Understanding how the spatial location of new roads and calmed areas affect wildlife in developing principles for decision-making present important challenges for future research.

We restricted our analysis to investigating some simple principles about how planners can reduce the impact of roads on wildlife by considering the relative effects of road density and traffic volume. However, these are not the only tools available for reducing road impacts. Numerous other strategies are regularly employed to help reduce the risk of wildlife mortality on existing roads, including speed reduction measures, fencing, road crossings, culverts under roads, overpasses, lighting, signs, road threshold treatments and wildlife reflectors, all with varying degrees of success [16]. In Port Stephens, a combination of advisory speed reductions zones, fencing and koala crossings have been proposed as part of a koala plan of management for the local government area [44]. However, the relative effectiveness of strategies that aim to reduce mortalities on existing roads compared to strategies focused on road network design remains uncertain. Addressing this would require not only an analysis of the effectiveness of each strategy, but also an estimation of the costs of implementation to identify the most cost-effective approach to reducing wildlife mortality [72], [73]. An important area for future work is therefore to incorporate costs into models of the effectiveness of alternative strategies for mitigating road mortalities, so as to identify investment priorities that achieve mortality reductions for lowest cost.

The impact of roads on wildlife populations arises from a range of complex spatial processes involving interactions between movement behaviours and the spatial pattern of habitat and roads. This study makes an important contribution to understanding how best to accommodate future increases in vehicle numbers and presents a coherent approach for doing this. One of the key challenges now is developing ways to effectively integrate the results of studies such as this into strategic planning processes for infrastructure and wildlife management [74]. This is challenging, but vital for biodiversity conservation.
